# The role of trust and habit in the adoption of mHealth by older adults in Hong Kong: a healthcare technology service acceptance (HTSA) model

**DOI:** 10.1186/s12877-023-03779-4

**Published:** 2023-02-04

**Authors:** Justina Yat Wa Liu, Golam Sorwar, Mohammed Sajedur Rahman, Md Rakibul Hoque

**Affiliations:** 1 School of Nursing, The Hong Kong Polytechnic University, Hong Kong, 999077 China; 2 Research Institute for Smart Ageing, The Hong Kong Polytechnic University, Hong Kong, 999077 China; 3 Faculty of Science and Engineering, Southern Cross University, Gold Coast, QLD 4225 Australia; 4 School of Business, Emporia State University, Emporia, KS 66801 USA; 5 Department of Management Information Systems, University of Dhaka, Dhaka, 1000 Bangladesh

**Keywords:** mHealth, UTAUT2, Older adults, Health technology service acceptance

## Abstract

**Background:**

Evidence from the literature suggests that mobile health (mHealth) services can potentially improve healthcare outcomes among older adults. Hence, the government of Hong Kong has recently taken several community and information technology (IT) services initiatives to train older adults on how to enhance their abilities and interest in using mHealth technology. Although mHealth services have been widely implemented globally, their adoption and use by older adults are very low, including those in Hong Kong. This study aims to understand key factors influencing mHealth use intention among the older Chinese population in Hong Kong.

**Methods:**

We extended the Unified Theory of Acceptance and Use of Technology (UTAUT2) as the basis of our conceptual framework. We applied Partial Least Squares path modeling method to conduct the Structural Equation Model (SEM) technique that allows measuring the theoretical validity of any conceptual framework. Convenience and snowball sampling methods were used to recruit participants aged 65 or above. In total, 201 valid responses were used for testing the theoretical validity of the proposed conceptual framework.

**Results:**

The primary finding shows that the widely used UTAUT2 model falls short in explaining mHealth service acceptance behavior in the Chinese older population in Hong Kong. We further propose a simplified model, the Healthcare Technology Service Acceptance (HTSA) model, to understand the formation of mHealth service acceptance behavior. The findings show that trust is an important component of technology service acceptance intention behavior that was missing in the UTAUT2 model. The results also show that several antecedent factors (i.e., social influence, government policy, and service quality) are critical in forming technology trust beliefs.

**Conclusions:**

The study shows that the HTSA model can better explain mHealth acceptance behavior than the UTAUT2 model. This study advances knowledge in the mHealth technology adoption domain by proposing a simplified new version of the UTAUT2 model for understanding healthcare technology service acceptance and use intention among older adults. The findings of the study provide valuable information to the Hong Kong government and healthcare organizations for wider adoption of mHealth services, especially in older adults.

**Supplementary Information:**

The online version contains supplementary material available at 10.1186/s12877-023-03779-4.

## Background

In recent decades, we have been experiencing a demographic shift due to increased life expectancy and a sharp decline in birth rates. Although these are clearly as a result of major achievements of modern sciences and improved healthcare, a new set of challenges have emerged in recent years regarding the independent living of older adults. Governments of various countries are also reportedly working to address challenges due to the aging population. Ensuring adequate quality of care for older adults and managing their health, therefore, pose a challenge for many governments.

Technology, such as mobile health (mHealth), has the potential to provide solutions for various health problems suffered by older adults [1]. Rapid advances in mobile and telecommunication technologies worldwide brought new opportunities, especially in delivering healthcare services to remote, elderly, and mobile-impaired populations. mHealth is defined by World Health Organization (WHO) as “medical and public health practice supported by mobile devices, such as mobile phones, patient monitoring devices, personal digital assistants (PDAs), and other wireless devices” [2]. The mHealth technology allows convenient and ubiquitous access to health services and health-related information at an affordable low cost [3]. In addition to the low-cost healthcare services, there is growing evidence that mHealth services facilitate improved overall health and wellbeing of older adults [3, 4]. Applications of mHealth technology have increased recently following the COVID-19 pandemic, which saw older adults be most impacted among all others [5].

Although various mHealth technologies evolved over time to support an aging population in place, existing literature suggests that there is only low-level acceptance of these technologies among this population group [6]. Our review of extant literature reveals a large body of literature that is focused on understanding the underlying mechanism and interplay of various factors influencing the use of mHealth technology by older adults across different geographical regions. A closer look at healthcare technology adoption literature reveals a wide range of factors contributing to the low acceptance. For example, fear of technology, technophobia among older adults, dependence on family support for technology adoption and usage, and the associated cost of using these platforms [7] prevent older adults from utilizing services using mobile technologies. Others have reported that personal attributes, such as self-efficacy, anxiety, social influence, training, and encouragement, have influence on healthcare technology usage among older adults [8]. A large group of researchers have reported usability factors, such as usefulness, ease of use, compatibility, intuitiveness of graphical user interfaces, performance expectancy, effort expectancy, and resistance to change [9, 10], are some of the major factors that contribute to the likelihood of mHealth technology usage by older adults. Moreover, aging has been found to be negatively related to the perceived ease of use and perceived usefulness of technology, two significant predictors of technology acceptance, which could represent a barrier to the adoption of mHealth by older adults [10].

In addition to the various factors mentioned above, some studies suggest that national and government health policies may have important influence on users’ perceptions of the health system and their actual use. For example, better management, organization, and effective use of resources through national health policies can strengthen the health system and enhance the quality of healthcare delivery [11]. Studies also show that government funding, plans, and policies have significant impacts on the wider adoption of mHealth services [11, 12]. Furthermore, some studies suggest that technology trust is an important factor that seems to have significantly influenced on the intention to use mHealth services by older adults [13].

More attention is needed to explain the factors that influence technology adoption by older adults, particularly because of conflicting findings. Researchers often extend the existing technology acceptance models, such as the unified theory of acceptance and use of technology (UTAUT) [14] or extended UTAUT (UTAUT2) [15] model, to explain mHealth acceptance behavior. However, despite many studies, there is still a lack of empirical studies testing the influence of government health policies on health technology, especially mHealth acceptance and usage. Typically, when explaining mHealth acceptance behavior, the UTAUT2 model does not consider the influence of government health policies and trust and their antecedents. Hence, we argue that developing a new model and comparing its ability to explain mHealth acceptance behavior with regard to the UTAUT2 model is necessary.

Therefore, the objectives of this study are threefold: first, to check the adequacy of the UTAUT2 model in explaining mHealth technology adoption among older adults; second, to extend the UTAUT2 model with additional variables such as Trust and Government health policy if the extension improves the predictive power of the UTAUT2 in the Hong Kong Context; finally, to further propose and test a simplified health technology service acceptance (HTSA) model to better understand factors that are behind the process of healthcare technology services acceptance.

When experimenting with the data, we first applied the original UTAUT2 model in the context of mHealth adoption by the older Hong Kong population. We then extended the UTAUT2 model by including three additional constructs (Government Health Policy, Service Quality, and Trust). After analyzing the results from the first two models, we finally proposed a simplified model that we believe has better explanatory power of mHealth services adoption intention by older adults in Hong Kong.

## Methodology

### Theoretical models and hypotheses

While some theories, such as the technology acceptance model (TAM) and theory of reasoned action (TRA), laid the foundation for understanding users’ technology acceptance behavior, the UTAUT2 is widely recognized as one of the leading theoretical models with its strong empirical support for the acceptance of various forms of technology by end-users. Given the widespread and global acceptance of UTAUT2, this study is grounded in this theoretical model. Individuals’ intended use provides insights into users’ technology adoption behavior. In existing technology adoption literature, intentions are assumed to predict individuals’ actual behavior [16] and have direct impact on actual technology use [17]. Some researchers, such as Hennington & Janz [18], have shown a direct relationship between intention and their actual technology use. As intended use helps to explain underlying reasons for the adoption, success or failure of a specific technology, some studies suggested that more focus should be given to understanding users’ perceptions and attitudes in relation to different technology adoption and use [19]. Moreover, many information system (IS) researchers (see for example [20]:) supported the use of intended use as a proxy for actual technology use behavior. Therefore, following this IS research trend and the suggestion of many IS researchers, we used intended behavior as a proxy for actual use behavior.

The original UTAUT [14] theory was incepted in 2003 to explain users’ intention to adopt new technology and their subsequent use behavior. According to UTAUT, the core constructs that influence individuals’ intention to use technology are performance expectancy, effort expectancy, and social influence [14]. UTAUT also asserts that facilitating conditions are important in forming individuals’ actual use behavior, which together with intention, can explain the actual technology use behavior [14]. This unified view was later extended to UTAUT2 by including three additional factors – hedonic motivation, price value, and habit for enhancing understanding of both behavioral intention and the actual use behavior.

**Fig. 1:**
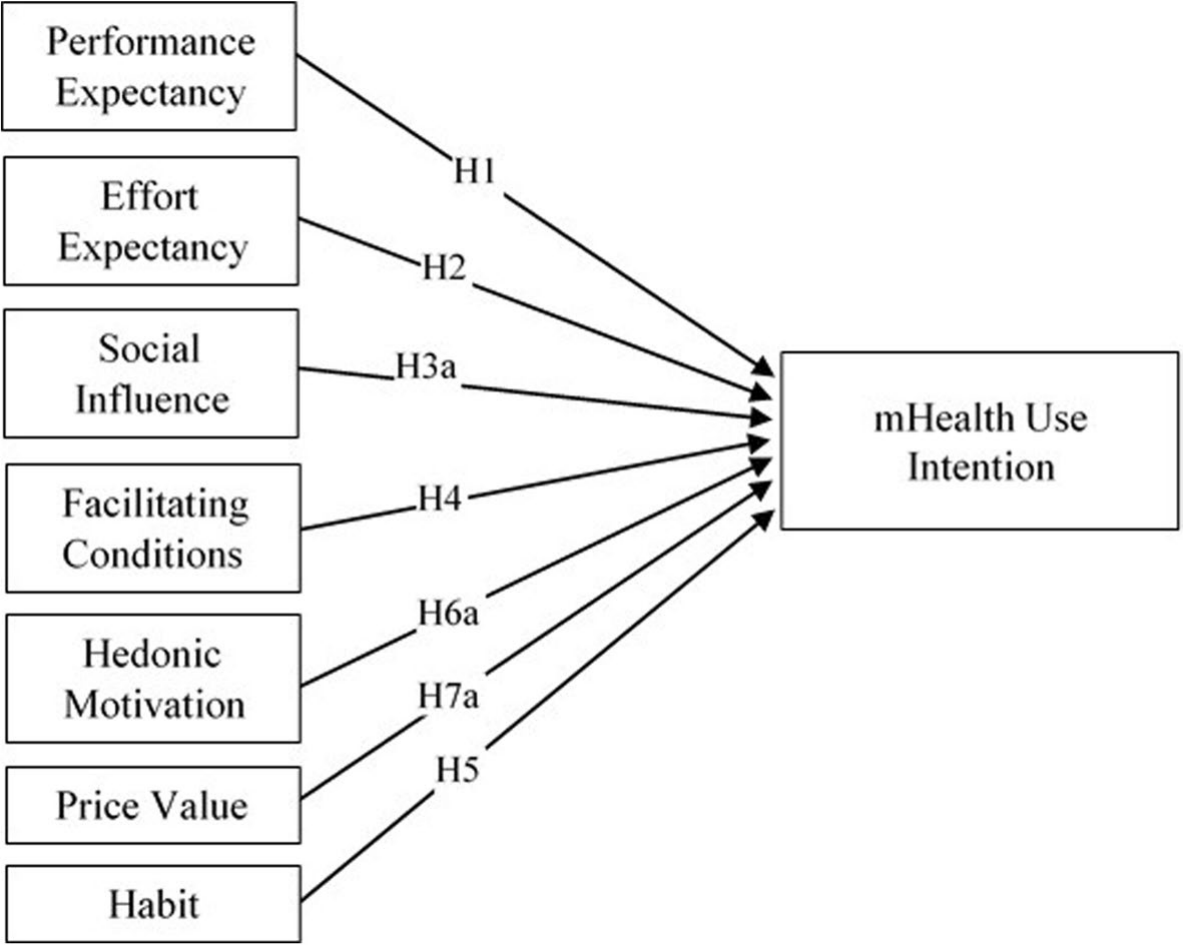
Research Model 1 - UTAUT2 [23]

Since its inception, UTAUT-based research has thrived and made significant impact in both research and practice in the past decade. A review of extant literature suggests the utilization of UTAUT theory in a wide area of research, including healthcare settings [21] as well as many other areas of technology use. Despite the wide use of UTAUT in information systems (IS) research, there are several limitations with UTAUT in explaining individuals’ technology adoption behavior regardless of technological and demographical contexts. For example, the UTAUT model does not consider cultural factors even though some studies (see for example [22]:) suggest a significant role of culture on technology adoption. In addition, UTAUT theory does not take into consideration the aspect of trust in determining behavioral intention and technology use behavior formation. Noting some of these limitations with the utilization of UTAUT, Venkatesh et al. [23] called for a paradigm shift of UTAUT extensions in technology acceptance and use. The authors of UTAUT further recommended the use of this model using the theoretical notion of contextualization because “context has become one of the important theoretical lens” in IS research [23]. Thus, in this study, we grounded our foundation using the UTAUT2 (Fig. 1) and contextualized the theory in the context of mHealth services use by older adults in Hong Kong*.*

#### Performance expectancy

Performance expectancy (PE) refers to “the degree to which an individual believes that using the system will help him or her to attain gains in performance” [14]. In the context of this study, we define PE as the degree to which an individual believes that using mHealth services will enhance the condition of his or her health. PE has been found as one of the strongest predictors [9, 15, 21] of individuals’ behavioral intention (BI) to adopt and use technology, including new healthcare related technologies such as mHealth. Therefore, we proposed the following hypothesis.

H1: Performance expectancy is positively associated with an individual’s behavioral intention to use mHealth services.

#### Effort expectancy

Venkatesh et al. [14] defined effort expectancy (EE) as “the degree of ease associated with the use of the system”. We define EE in the context of this study as the degree to which an individual believes that receiving mHealth services is easy without needing significant effort. EE has been reported to have a significant positive impact on technology, including healthcare technologies such as mHealth, use behavioral intention (see for examples [9, 15, 21]:). Based on these findings, we proposed the following hypothesis:

H2: Effort expectancy is positively associated with an individual’s behavioral intention to use mHealth services.

#### Social influence

Venkatesh et al. [14] defined social influence (SI) as “the degree to which an individual perceives that important others (e.g., family and friends) believe they should use the new system.” In this study, we define SI as the degree to which an individual believes that his or her decision to receive mHealth services is influenced by significant others. Existing research suggests SI is a significant contributor to individuals’ behavioral intention decision when pertaining to technology use, including mHealth technology (see for example [24]:). Therefore, we posited the following hypothesis:

H3a: Social influence is positively associated with an individual’s behavioral intention to use mHealth services.

H3b: Social influence positively influences an individual’s trust belief in mHealth services.

#### Facilitating conditions

In the original UTAUT study, Venkatesh et al. [14] refer to facilitating conditions (FC) as “the degree to which an individual believes that an organizational and technical infrastructure exists to support the use of the system”. To fit the context of this study, we defined FC as the degree to which an individual believes they have the necessary knowledge, resources, and supports for using mHealth services. FC is a determinant in BI for using different technologies, including healthcare technologies such as mHealth (see for example [14]:). However, Venkatesh et al. [14] stated that the influence of facilitating conditions on behavioral intention diminishes and it is non-significant in predicting intention if both performance expectancy and effort expectancy is present in the model. Other studies (see for examples [9, 25]:) also found non-significant relationships between these two constructs in the presence of performance expectancy and effort expectancy. Thus, we proposed the following hypothesis regarding the relationship between facilitating conditions and behavioral intention to use mHealth services.

H4: Facilitating conditions do not have a positive influence in determining behavioral intention to use mHealth services.

#### Habit

Venkatesh et al. [15] described habit (HA) as “the extent to which people tend to perform behaviors automatically because of learning”. In the context of this study, we define habit as an individual’s belief about his or her frequent use of mHealth services and one’s natural reliance on this technology for healthcare needs. Existing research (see for examples [14, 26, 27]:) suggests that consumers’ habits play a significant impact on technology use, both directly and as a behavioral intention path to affect their behavior. As it can be safely assumed that “habitual previous behavior in a given context will predict behavioral intentions in the same context” [28], the following hypothesis was proposed.

H5: Habit is positively associated with an individual’s behavioral intention to use mHealth services.

#### Hedonic motivation

Venkatesh et al. [15] defined hedonic motivation (HM) as “the fun or pleasure derived from using technology”. Research has shown strong relationship between this construct and behavioral intention to the acceptance and use of technology, including healthcare technology such as mHealth. Therefore, relying on the evidence from existing literature, we posited the following hypothesis:

H6a: Hedonic motivation is positively associated with an individual’s behavioral intention to use mHealth services.

Hedonic motivation is also known to influence people’s habits. Although most technology adoption studies suggest a strong relationship between hedonic motivation and intention to use technology, a few studies investigated the interrelationship between hedonic motivation and habit. Habit is found as a mediator between hedonic motivation and intention to use technology [29]. Furthermore, Chiu [30] found that hedonic motivation leads to the building of habit of technology use. Based on this evidence in existing literature, the following hypothesis was posited.

H6b: Hedonic motivation is positively associated with habit in the use of mHealth services.

#### Price value

Price value (PV) is an outcome of a cost-benefit decision as individuals go through a process of cost and benefit analysis to gauge their perception about the value of certain products or services. The tradeoff between consumers’ benefits (i.e., efficiency, convenience, quality, etc.) and costs (i.e., monetary expenses, the difficulty of use, sacrifice, etc.) perceptions determine their value perceptions, which then further influence their decision. Thus, researchers defined perceived value as “consumers’ cognitive tradeoff between the perceived benefits of the applications and the monetary cost for using them” [15]. In this study, we define perceived value as the degree to which an individual believes that the use of mHealth services will provide more benefits than the costs of using the technology. A number of studies have shown that users’ perception of value derives from people’s intention to use and continue to use technologies and technological services (see for example [15]:). As mHealth is a cost-effective medium of receiving healthcare service, the researchers infer that it is a strong determinant of behavioral intention to continue to use the technology [26]. Thus, the following hypothesis was posited.

H7a: Price value is positively associated with an individual’s intention to use mHealth services.

Although no studies to date show a direct relationship between price value and habit, studies on the formation of people’s habit suggest that people’s habit can be influenced by increasing or decreasing value or reward. For example, Loewenstein et al. [31] reported that increasing incentives help in forming habits with regards to healthy food consumption. Thus, we hypothesized the following relationship between price value and habit.

H7b: Price value is positively associated with an individual’s habit of using mHealth services.

#### Trust

Technology trust is defined as “the belief that specific technology has the capability, functions, or features to do for one what one needs to be done” [32]. In the context of this study, we define technology trust (TR) belief as the degree to which an individual believes that mHealth technology has the capability to provide adequate and responsive help to fulfill their healthcare needs. Technological trust is even more important in healthcare as more and more healthcare services are being delivered using technology that requires patients’ interaction, engagement, and disclosure of their sensitive health and personal information. Lack of trust in healthcare technology can result in many adverse effects on patients’ health. Zulman et al. [33] studied technology trust and the use of healthcare resources by older adults and reported that distrust of Internet technology is responsible for avoiding the use of technology as a health resource. Greater Trust is believed to affect behavioral intention positively, especially in behavioral intention to use healthcare technologies [34]. Therefore, we proposed the following hypothesis:

H8: Technology trust is positively associated with an individual’s behavioral intention to use mHealth services.

As mHealth services are provided and received remotely, many factors can hinder or facilitate their successful delivery and reception. For example, the quality of service delivered is often time and serves as a factor that helps to gain trust, which in turn determines continuous use of the technology. In addition, the use of mHealth services requires clients to disclose and exchange their personal and sensitive data over the Internet using different devices, such as tablets, cellphones, etc. The concern for security and privacy is an important factor in situations where data are exchanged between hosts and clients. Thus, given the complex nature of the trust construct, it is imperative that we dig deeper to understand how individuals’ perceptions are formed for this construct. Thus, we include two additional constructs (Service Quality and Government Policy) that will serve as antecedent factors. We believe these two additional factors will provide important insights into the formation of mHealth services users’ trust beliefs.

#### Service quality

Although delivering quality service has been an essential component in the success of technology adoption, the construct service quality (SQ) has found little attention in IS literature. The prevalent use of IT for improving customer satisfaction garnered much attraction on IT service quality in recent years. The perception of service quality is the consequence of individuals’ evaluation of the quality of service received using technology. This construct has been extensively studied in marketing as well as consumer behavior literature, where service quality is commonly defined as individuals’ judgement about the overall superiority of service experience [35]. In the context of this study, we define service quality perception as the degree to which mHealth services can meet the needs of its users. Several studies [36] reported indirect relationships where service quality influences satisfaction, which in turn influences behavioral intention. Perceived service quality has also been studied in the healthcare context. For instance, Akter et al. [37] studied mHealth continuance intention in Bangladesh and found that service quality perception significantly influences the continuance of mHealth services use. Based on the evidence, we posited the following hypothesis.

H9a: Service quality perception positively influences an individual’s behavioral intention to use mHealth services.

Individuals often rely on their experience with the quality of service they receive to gain a level of trust in the technology. Positive service quality experience increases their trust, whereas negative experience with service quality lowers their trust toward the technology. Existing literature also suggests a strong relationship between perceived service quality and individuals’ trust beliefs. In the context of healthcare, Akter et al. [37] studied mHealth services continuance use behavior and found that service quality not only influences behavioral intention but also influences trust beliefs, which in turn influences individuals’ mHealth services continuance intention. Thus, based on these findings, the following hypothesis was proposed.

H9b: Perceived service quality positively influences an individual’s trust belief.

#### Government policy

Government policy (GP) is known to shape the direction of citizens’ product use. For example, if the government of a country imposes sanctions on using a certain type or brand of technology (e.g., US sanctions on using Huawei devices), it will prevent citizens from using that technology. On the other hand, if the government passes laws and regulations to promote certain product or services, citizens will find it easy to use them. Thus, government policies play a critical role in diffusing certain technology in society by making favorable policies and environments. In investigating technology adoption issues, researchers suggest that various government policies play an important role in influencing citizens’ technology adoption. A recent study by Wang et al. [38] reports that government policy, such as use promotion, is one of the most important factors in users’ continuance intention in using technology. Llewellyn et al. [39] reported that healthcare policies act as a significant barrier or facilitator for promoting hospital and community-based services. Many studies recommended that government policymakers pass policies to increase mHealth adoption. For example, Hoque et al. [9] and others proposed to make government policies to maximize mHealth services adoption. Based on this evidence, we posited the following hypothesis:

H10a: Favorable government policy positively influences an individual’s behavioral intention to use mHealth services.

Government policy is also known to influence people’s trust in technology or service. Government policy often shapes whether individuals view certain technology or service as safe or risky. Especially if the technology requires people to disclose their sensitive personal information. In studying human resource information systems, Lippert & Swiercz [40] suggest that policy is an important factor in establishing technology trust, and the level of trust among users guide their decisions whether to use or not to use the technology. The absence of an appropriate policy leads to uncertainties that motivate individuals to avoid technologies. Lu et al. [41] studied facilitating conditions of trust for wireless technology and found that policies play a key role in establishing users’ trust, which in turn influences intention to use the technology. Although we found no study examining the relationship between government healthcare policies and healthcare technology trust and technology adoption, given the sensitive nature of health information, we believe healthcare policies promoted by the government are a critical antecedent and have more impact on shaping users’ healthcare technology trust beliefs. Thus, we posited the following hypothesis.

H10b: Favorable government policy is positively associated with an individual’s trust belief.

In addition to the service quality and government policy, we believe social influence will also serve as an important antecedent factor for forming individuals’ trust beliefs toward mHealth services. Existing research suggests that social influence greatly influences individuals’ trust beliefs toward specific technologies (see for example [42]:).

**Fig. 2:**
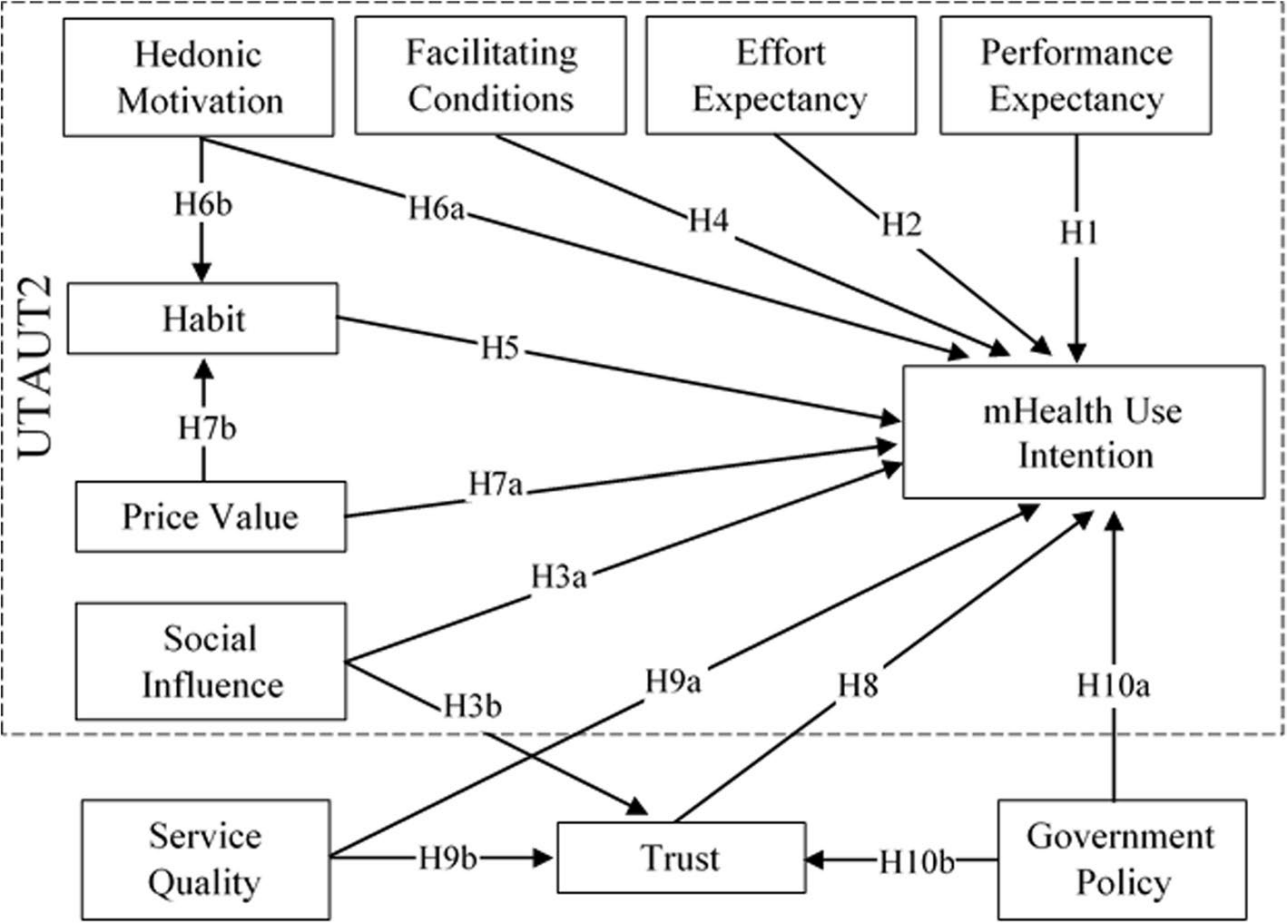
Research Model 2 – Extended UTAUT2 Model

**Fig. 3:**
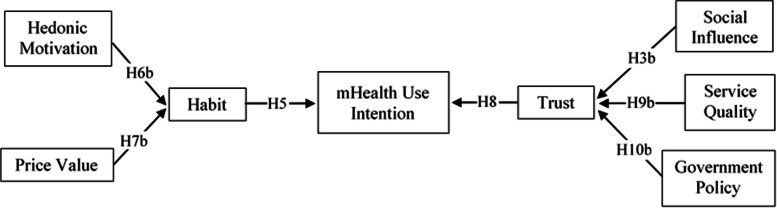
Research Model 3 – Health Technology Service Acceptance (HTSA) Model

Figure 2 shows the extended UTAUT2 model that we propose as the second research model for this study. Based on the findings of our first two (UTAUT2 and Extended UTAUT2) research models, we propose the third simplified research model for this study. We refer to the simplified model as Health Technology Service Acceptance (HTSA) model, shown in Fig. 3, with the following hypotheses.

H3b: Social influence positively influences an individual’s trust belief in mHealth services.

H5: Habit is positively associated with an individual’s behavioral intention to use mHealth services.

H6b: Hedonic motivation is positively associated with Habit in the use of mHealth services.

H7b: Price value is positively associated with an individual’s habit in using mHealth services.

H8: Technology trust is positively associated with an individual’s behavioral intention to use mHealth services.

H9b: Perceived service quality positively influences an individual’s trust belief.

H10b: Favorable government policy is positively associated with an individual’s trust belief.

### Instruments development and pretest

To ensure the validity of all measures, the measurement items (see Additional file 1) for latent constructs within the proposed model were developed from prior studies. Items for PE, EE, SI, FC, HM, PV, and HA, were derived from Dwivedi et al. [43], Venkatesh et al. [14] and Venkatesh & Davis [24]. TR was derived from Fischer et al. [44]. Items for SQ were derived from Akter et al. [37] and items for HP from Llewellyn et al. [39]. A structured questionnaire was originally developed in English by all authors and then translated into Chinese.

First, the original English version was translated into Chinese by a professional translator a bilingual speaker of native Chinese and English. Then, the first author (JL), who have doctor degree in nursing and is in native Chinese and English, backward-translated the draft into English. Finally, the backward-translated English version was compared with the original English scale by the two translators to reach a consensus on the Chinese version questionnaire. An expert panel, with an excellent command of the English language and good knowledge of mHealth, was then set up for the determination of the content validity index (CVI) of the translated Chinese questionnaire. Each question was rated on a 4-point Likert scale, with 4 as most relevant. Any item in the Chinese questionnaire with a score below 3.5 was modified based on the panel’s recommendations until the score was above the desired mean score of 3.5. These modifications were done a maximum of three times for only a few items. We also had a professor from the language department ensure the accuracy between English and Chinese versions. All items were rated using a five-point Likert scale, ranging from “strongly disagree” to “strongly agree”.

### Data collection procedure

Convenience and snowball sampling methods were used to recruit older adults aged 65 or above who were able to read and communicate in Chinese. They had to be living in Hong Kong for at least 7 years. Older adults were excluded if they had cognitive impairment and / or physical impairment (such as blindness) that rendered them unable to use smartphones and mobile apps. Accommodating a diverse group of older adults, this study recruited participants with different levels of experience using mobile apps on smartphones regardless of their dependency on others to use the technology.

Ethical approval for this study was obtained from The Human Subjects Ethics Committee of The Hong Kong Polytechnic University. Permission to conduct the study was also sought from the person in charge of the community center for older adults. Flyers introducing the aims of this study were posted at the centers. Older adults who wanted to join the survey were enrolled through the centers. According to sample selection criteria, the eligibility of those wanting to participate in this study was assessed by a well-trained research assistant (RA). Written informed consent was obtained from each participant before they took part in a structured face- to- face interview. All of the interviews were conducted by the RA according to a structured interview guideline developed by the principal investigator.

Participants could also complete the survey through an online platform – mySurvey. Those eligible and interested in participating completed an online informed consent document and proceeded to the questionnaire. Data were collected anonymously. IP addresses were used to identify and eliminate potential duplicate entries from the same user.

### Demographic profiles

A total of 201 valid responses (among them 47 online and 154 paper based) were used for testing the proposed model. The sample size requirements may differ in different types of statistical analysis, and a variety of opinions were also observed in the literature even when applying the same tools. In SEM-PLS reference for determination of sample size, many scholars recommend a sample size equivalent to 10 observations per model construct. There are 11 constructs in this study. The recommended sample size for this study should be more than 110. The sample size calculation for conducting the structural equation model (SEM) shows that the recommended sample size for this study is 195. In the calculation, the expected effect size was 0.3, the number of latent variables was 11, the number of observed variables was 44, the *p*-value was set as 0.05, and the statistical power was 0.8. So, the sample size of 201 in this study was more than the recommended sample size of 195. The demographic characteristics of respondents are presented in Table 1. Most respondents were between the age of 65–69 years old (45.27%) or 70–74 years old (36.8%). A total of 65.17% of the sample was female. Although all participants used a smart phone, only *n* = 29 (14.4%) indicated that they had used health applications related to nutrition advice, fall prevention, fitness, etc.

**Table 1:** Demographics of respondents

Variable	Frequency	Percentage
Gender		
Male	70	34.8%
Female	131	65.2%
Age		
60 to 64	0	0.0%
65 to 69	91	45.3%
70 to 74	74	36.8%
75 to 79	18	9.0%
80 and above	18	9.0%
Educational Qualifications		
None	3	1.5%
Primary	40	19.9%
Secondary	38	18.9%
Higher Secondary	87	43.3%
Bachelor’s Degree	24	11.9%
Master’s Degree	6	3.0%
Doctorate	1	0.5%
Others	1	0.5%
Marital Status		
Single or never married	32	15.9%
Married or in a partnership	137	68.2%
Widowed	24	11.9%
Divorced or separated	8	4.0%

## Results

SmartPLS software was used for conducting structural equation modeling (SEM) using the partial least squares (PLS) path modeling method [45]. This method allows estimating the theory-based framework proposed in this study by measuring the latent variables at the observation level (i.e., measurement model) and testing the relationships between the latent variables on the theoretical level (i.e., structural model). First, we conducted a measurement model analysis to ensure the validity and reliability of the instrument. Then, we conducted the structural model analysis to test our hypotheses. In this section, we present our results from both analyses.

### Measurement model

According to Hair et al. [45], the measurement model needs to be assessed for internal consistency reliability, discriminant validity, and convergent validity to confirm the model fit. In this study, Cronbach’s alpha (α), Dijkstra-Henseler’s rho, and composite reliability were used to evaluate the internal consistency reliability. All constructs had Cronbach’s alpha ranging from 0.842 to 0.950, composite reliability values ranging from 0.895 to 0.968, rho values ranging from 0.853 to 0.951 that are well above 0.7, a minimum recommended threshold for both of these measurement criteria [45]. Thus, based on measures provided in Table 2, all constructs have high internal consistency reliability.

**Table 2:** The measurement model

Constructs	Items	Loadings	Cronbach’s alpha (α)	Rho_	CR	AVE	VIF
Use Intention	CI1	0.955	0.950	0.951	0.968	0.910	5.499
	CI2	0.961					5.969
	CI3	0.945					4.305
Performance Expectancy	PE1	0.718	0.896	0.905	0.920	0.658	1.991
	PE2	0.833					2.687
	PE3	0.855					2.591
	PE4	0.861					2.711
	PE5	0.770					2.113
	PE6	0.821					2.347
Effort Expectancy	EE1	0.907	0.911	0.925	0.937	0.788	3.429
	EE2	0.871					2.244
	EE3	0.904					3.443
	EE4	0.867					2.788
Facilitating Conditions	FC1	0.825	0.842	0.853	0.895	0.683	2.061
	FC2	0.875					2.758
	FC3	0.885					2.568
	FC4	0.707					1.498
Social Influence	SI1	0.885	0.921	0.922	0.944	0.810	3.220
	SI2	0.909					3.336
	SI3	0.940					4.917
	SI4	0.863					2.561
Habit	HA1	0.927	0.922	0.924	0.950	0.865	3.192
	HA2	0.921					3.333
	HA3	0.941					3.968
Hedonic Motivation	HM1	0.945	0.929	0.937	0.955	0.876	4.538
	HM2	0.958					5.410
	HM3	0.903					2.915
Price Value	PV1	0.878	0.902	0.909	0.939	0.837	2.328
	PV2	0.936					3.655
	PV3	0.929					3.361
Trust	TR1	0.899	0.935	0.938	0.953	0.836	3.188
	TR2	0.888					3.071
	TR3	0.945					6.375
	TR4	0.924					5.256
Service Quality	SQ1	0.823	0.939	0.949	0.952	0.768	2.544
	SQ2	0.879					3.500
	SQ3	0.916					4.538
	SQ4	0.866					3.020
	SQ5	0.899					3.752
	SQ6	0.872					3.148
Government Policy	GP1	0.925	0.940	0.941	0.957	0.848	4.500
	GP2	0.926					4.705
	GP3	0.925					4.165
	GP4	0.908					3.515

*CR* Composite Reliability, *AVE* Average Variance Extracted

We confirmed the convergent validity of our instrument using factor loadings and latent variables average variance extracted (AVE) values. While factor loadings are recommended to be higher than 0.6 to ensure the convergent validity of the instrument, the AVE values are recommended to be higher than 0.5. Table 2 shows that the factor loading values for items and the AVE values for each construct were much higher than the recommended thresholds, confirming a strong convergent validity of our measurement instrument.

The discriminant validity of the model was examined by following Chin’s [46] recommendation to use the square root of the AVEs. To have a satisfactory level of discriminant validity, the square root of AVE should be higher than its correlation with other constructs in the model. The Fornell-Larcker criteria analysis is often used for discriminant validity, where the diagonal values need to be higher than the elements in the corresponding rows and columns to satisfy the conditions for discriminant validity. The results in Table 3 reveal that the measurement instrument used in this study sufficiently met the criteria of discriminant validity. We used another measuring criterion, the heterotrait-monotrait ratio of correlations (HTMT), to ensure the discriminant validity of our model. Our results show HTMT values ranging between 0.319 and 0.806 that are much lower than the recommended threshold of one [47].

**Table 3:** Fornetll-larcker criteria analysis

	CI	EE	FC	GP	HA	HM	PE	PV	SQ	SI	TR
CI	0.954										
EE	0.531	0.887									
FC	0.545	0.616	0.826								
GP	0.453	0.308	0.288	0.921							
HA	0.755	0.530	0.538	0.399	0.930						
HM	0.588	0.553	0.642	0.370	0.648	0.936					
PE	0.547	0.574	0.452	0.535	0.539	0.527	0.811				
PV	0.549	0.422	0.406	0.502	0.595	0.482	0.499	0.915			
SQ	0.658	0.428	0.560	0.527	0.629	0.624	0.468	0.588	0.876		
SI	0.558	0.503	0.477	0.498	0.549	0.628	0.552	0.577	0.590	0.900	
TR	0.550	0.360	0.368	0.521	0.441	0.421	0.466	0.534	0.626	0.561	0.915

*CI* Continuance Intention, *EE* Effort Expectancy, *FC* Facilitating Conditions, *GP* Government Policy, *HA* Habit, *HM* Hedonic Motivation, *PE* Performance Expectancy, *PV* Price Value, *SQ* Service Quality, *SI* Social Influence, *TR* Trust

We examined the model fit using the results from bootstrap-based statistical analysis. We used the only approximate model fit criterion implemented for PLS path modeling, standardized root means square residual (SRMR). A cut-off value of 0.08 indicates an acceptable fit for the model. Results from our bootstrap-based test show an SRMR value of 0.044, which is much lower than the recommended acceptable value for a good model fit. Therefore, our measurement model has overall goodness of fit.

We have also tested the multicollinearity and common method bias, two common issues with any survey method study. We used the variance inflation factor (VIF) values as shown in Table 2 to rule out the multicollinearity issue. All but three items have VIF values that are much lower than the recommended maximum threshold of 5, indicating that multicollinearity is not an issue in our measurement instrument. In order to rule out common method bias, we conducted the Harman single-fact test and found that no single factor is apparent in the un-rotated factor structure, which is an indication that common method bias is not an issue in this study.

### Structural model and hypothesis testing

We tested the hypotheses using three different models. First, we tested the original UTAUT2 model (Fig. 1) to validate the applicability of the model in the healthcare context. Second, we tested using our proposed extended UTAUT2 model (Fig. 2) with trust and antecedents of trust. Third, after evaluating the results of the second model analysis, we re-tested the data using our proposed simplified HTSA model (Fig. 3). To identify the hypothesized relationships among the factors in the study, the structural model was tested by path coefficients (β), t-statistics, and *p*-values, presented in Table 4.

**Table 4:** PLS-SEM path analysis results summary for structural model

Hypotheses		Model 1 (UTAUT2)			Model 2 (Extended UTAUT2)			Model 3 (HTSA)		
		β	T Stat.	Support	β	T Stat.	Support	β	T Stat.	Support
H1	PE → CI	.095	1.528	No	.055	0.718	No			
H2	EE → CI	.050	0.694	No	.062	0.606	No			
H3a	SI → CI	.098	1.028	No	.020	0.191	No			
H4	FC → CI	.112	1.436	Yes+	.086	0.610	Yes+			
H5	HA → CI	.513***	3.736	Yes	.572***	3.684	Yes	.683***	7.648	Yes
H6a	HM → CI	.011	0.127	No	- .070	0.572	No			
H7a	PV → CI	.071	0.830	No	- .060	0.596	No			
H8	TR → CI				.164*	1.967	Yes	.259*	2.415	Yes
H9a	SQ → CI				.145	1.214	No			
H10a	GP → CI				.032	0.351	No			
H10b	GP → TR				.199**	2.618	Yes	.200**	2.607	Yes
H9b	SQ → TR				.401***	3.926	Yes	.402***	3.979	Yes
H3b	SI → TR				.243*	2.346	Yes	.242*	2.349	Yes
H6b	HM → HA				.492***	6.066	Yes	.491***	6.133	Yes
H7b	PV → HA				.393***	4.609	Yes	.395***	4.781	Yes
Dependent Variables		R^2^	Adj. R^2^		R^2^	Adj. R^2^		R^2^	Adj. R^2^	
Use Intention		.628***	.614***		.725***	.710***		.701***	.698***	
Trust Belief					.525***	.517***		.525***	.518***	
Habit					.597***	.593***		.601***	.597***	

*CI* Use Intention, *PE* Performance Expectancy, *EE* Effort Expectancy, *SI* Social Influence, *FC* Facilitating Conditions, *HA* Habit, *TR* Trust, *HM* Hedonic Motivation, *PV* Price Value, *SQ* Service Quality, *GP* Government Policy

+ = Supported because H4 was hypothesized a non-significant relationship

^*^p < 0.05

^**^p < 0.01

^***^p < 0.001

The results for original UTAUT2 (Model 1) show that the latent variables: performance expectancy, effort expectancy, social influence, facilitating conditions, hedonic motivation, and price value, had no influence on individuals’ intention to use mHealth services. On the other hand, only the latent variable, habit, was found to have significant and strong influence on the intention to continue to use mHealth services.

**Fig. 4:**
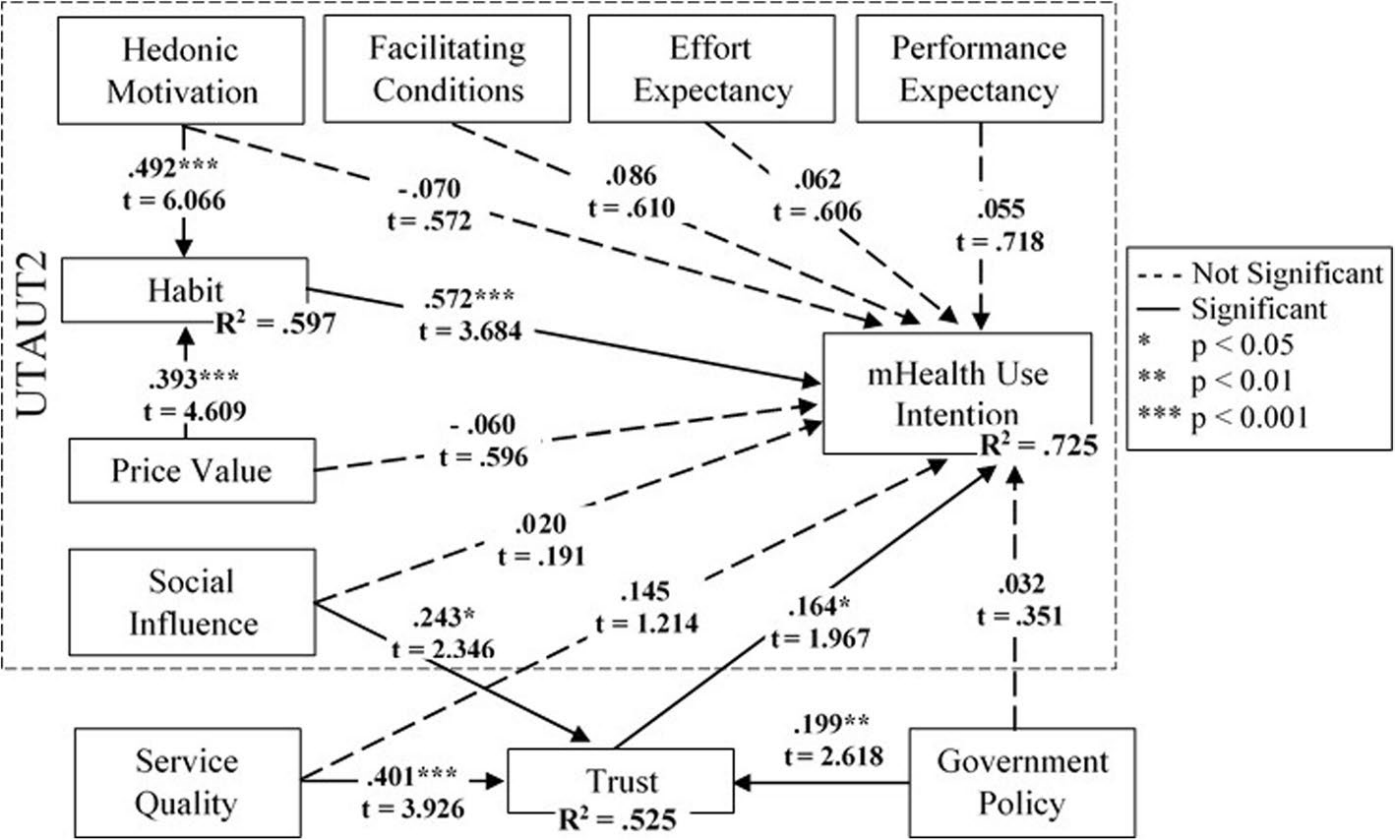
Path Significant Results for Extended UTAUT2 Model

We then performed the same analysis using the extended UTAUT2 research model (Model 2). The results show that only habit from the original UTAUT2 model significantly influenced the intention to use mHealth services (β = 0.572, t = 3.684, *p* = 0.000), supporting H5. The results further show that the Trust also significantly influenced the intention (β = 0.164, t = 1.967, *p* = 0.049), supporting H8 in our extended research model. The results did not support the relationships between all other latent variables in the original UTAUT model and the dependent variable, intention. Thus, H1, H2, H3a, H6a, and H7a were not supported by model 2 analysis. The results show that the relationships between social influence and trust (β = 0.243, t = 2.346, *p* = 0.019); service quality and trust (β = 0.401, t = 3.926, *p* = 0.000); and government policy and trust (β = 0.199, t = 2.618, *p* = 0.009) were all significant, supporting H3b, H9b, and H10b. Similarly, the relationships between habit and its antecedent factors: hedonic motivation and price value (β = 0.492, t = 6.066, *p* = 0.000; β = 0.393, t = 4.609, *p* = 0.000, respectively) were both significant, supporting H6b and H7b. However, we found no support for service quality and government policy as antecedent factors for individuals’ intention to continue to use mHealth services. Thus, H9a and H10a were not supported. Figure 4 shows the path significant results for the extended UTAUT2 model.

**Fig. 5:**
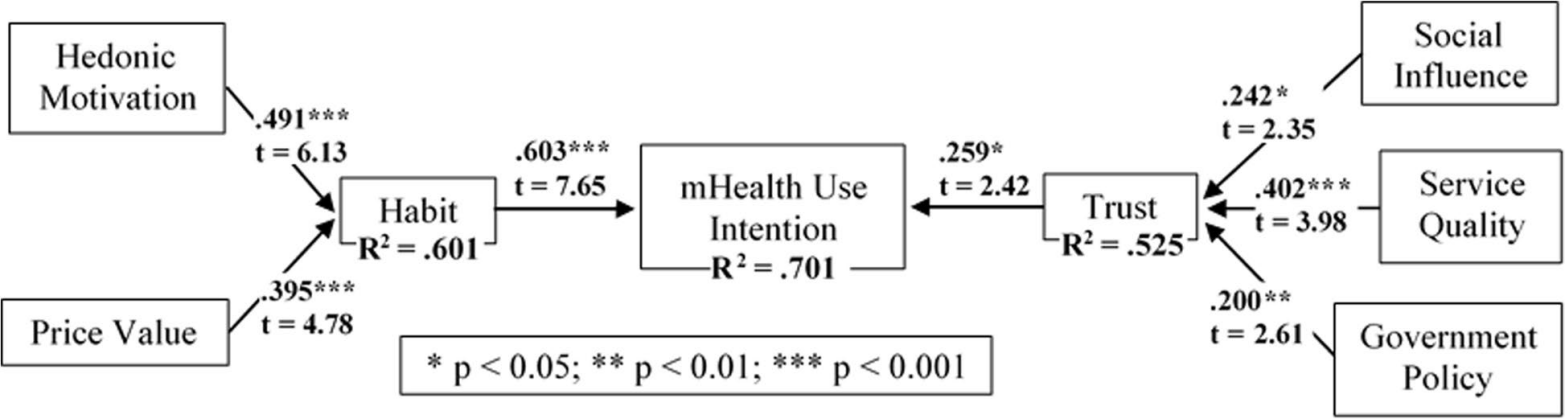
Path Significant Results for Simplified UTAUT2 – HTSA Model

Based on the findings from our first two model analyses, we tested our proposed HTSA model (Model 3). The results show that the relationship between both habit and intention (β = 0.683, t = 7.648, *p* = 0.000) and trust and intention (β = 0.259, t = 2.415, *p* = 0.016) were significant, supporting H5 and H8. The results further show the relationships between social influence and trust (β = 0.242, t = 2.349, *p* = 0.019); service quality and trust (β = 0.402, t = 3.979, *p* = 0.000); and government policy and trust (β = 0.200, t = 2.607, *p* = 0.009) were all significant, supporting H3b, H9b, and H10b. Finally, for the antecedents of habit, the results show that the paths between hedonic motivation and habit (β = 0.491, t = 6.133, *p* = 0.00) and between price value and habit (β = 0.395, t = 4.781, *p* = 0.00) were significant, supporting both s H6b and H7b. Therefore, all of the hypotheses in our proposed simplified HTSA model are found to be supported by our analyses. Figure 5 shows the path significant results for the proposed simplified UTAUT2 (HTSA) model.

## Discussion

This study sought to understand underlying mechanisms in the formation of the behavioral intention to use mHealth services by older adults. We laid out three specific goals for this study. First, we wanted to test the applicability of Venkatesh’s [15] UTAUT2 model in the healthcare domain, specifically to explain mHealth use behavior by older adults. Second, to fill a limitation of the UTAUT2 model by extending the model with the inclusion of a technology trust component. Third, to propose a simplified model that could be more appropriate for explaining healthcare technology use behavior.

The results suggest that six of the seven UTAUT2 core factors are not significant determinants of older adults’ intention to use mHealth in the HK context (Table 4), indicating that the UTAUT2 may not be reliable in explaining context-specific technology adoption and/or use behavior and results based on the model can vary greatly contextually. This inconsistency in results yielded by the UTAUT2 model is also reported in the meta-analytic study by Tamilmani et al. [48] when investigating the reliability of UTAUT2.

The analyses of our extended UTAUT2 model also reveal that six of the core factors are not significant determinants of older adults’ behavioral intention to use mHealth. These findings are consistent with the literature. For example, Alam et al. [34] found no significant relationship between effort expectancy, price value and intention in the Bangladesh context. The explanation of the weak influence of effort expectancy can be because the mHealth app interface is now more user friendly for older adults [34]. To further explain the weak influence of price value, we believe that it might be because of the fact that healthcare is a necessary need in people’s lives and when the need for caring for their health is the greatest concern for someone, the significance of price value becomes low.

Venkatesh et al. [14] reported that the influence of facilitating conditions on behavioral intention becomes insignificant if both performance expectancy and effort expectancy are present in a research model, which is in line with our finding for the relationship between facilitating conditions and intention.

We found that social influence and hedonic motivation are insignificant predictors of behavioral intention, also consistent with existing literature [43, 49]. It might indicate that older users are more concerned about the usefulness and benefits than fun and excitement about using the mHealth app.

The findings of our extended model (model 2) show significant relationship between trust and behavioral intention to use mHealth services. Among the three other factors added to the UTAUT2 model, service quality, social influence, and government policy were all found to be strong determinants of trust and insignificant determinants of intention, implying that trust is a stronger antecedent of behavioral intention than service quality, social influence, and government policy. Therefore, trust should be a mediating factor for the relationship between these three antecedent variables and behavioral intention. The strong relationship between service quality and trust is also supported by Cao et al. [50]. Previous studies also support our findings on the relationships between social influence and users’ trust [51] as well as the influence of government health policy on user trust [52]. Additionally, the findings of our study show a strong relationship between habit and its antecedents: price value and hedonic motivation, which is supported by previous studies (see for example [29]:).

Moreover, this study explored the role of trust in influencing the behavioral intention to use mHealth technology and found positive relationship consistent with existing literature (see for examples [33, 44]:). The findings of the present study in relation to the impact of habit on behavioral intention are consistent with past studies [15, 26].

Based on the findings in our second (extended UTAUT2) model, we proposed and tested a third model (HTSA). This model analysis revealed interesting findings compared to the previous two models. The empirical evidence of the original UTAUT2 model found only one out of seven antecedents for behavioral intention as significant with R-squared = 0.628. Our proposed HTSA resulted in a much better explanatory power. As the results for the extended model (Model 2) showed that only one of the factors from the original UTAUT2 was significant, our simplified model only kept the one factor from the original UTAUT2 model along with the new factor (technology trust belief) as antecedents for behavioral intention. Together, these two factors explain 70.1% (R-squared = .701) of the variances for use intention, showing a significant improvement from the original UTAUT2 model results. This high variance for trust also indicates that the extension of the UTAUT2 model by adding trust (TR) has increased the model fitness in ultimately explaining the variance in behavioral intention (BI) for the use of mHealth services by older adults. The extended model analysis further suggests antecedents for both habit and trust. The R-squared value of 0.525 for trust suggests that all the independent factors, social influence, service quality, and government policy, can explain up to 52.5% of the variance in trust, which ultimately influences the behavioral intention to use mHealth services. The R-squared value of 0.601 for habit further suggests that the two antecedent factors, price value and hedonic motivation, together explain 60% of the variances in forming a habit, which also in turn helps to explain better behavioral intention for using mHealth services.

### Implications

This study makes several key theoretical and practical contributions to technology adoption and diffusion domain.

#### Theoretical contribution

First, although the UTAUT2 model was developed by combining eight competing theories from the technology acceptance domain, the model lacks a vital component of technology adoption, which is technology trust. A large body of researchers agrees that one of the biggest motivators to utilize sensitive technologies (such as mHealth) is individuals’ trust in the specific technology. This study addresses this missing concept by extending the UTAUT2 model by incorporating trust and its antecedent factors. We show that the addition of the trust component significantly improves the explanatory power of the UTAUT2 model for technology adoption and further diffusion.

Second, this study also makes a significant theoretical contribution to the domain of technology adoption by proposing a simplified healthcare technology service acceptance (HTSA) model. Because “context has become one of the important theoretical lenses” in IS research [23], the authors of UTAUT further recommended using this model using the theoretical notion of contextualization. Responding to the call for contextualization of the UTAUT2 model, we believe that our simplified model is a step forward in the contextualization of the UTAUT2 model for understanding the use behavior of mHealth services by older adults.

Third, the applicability of the UTAUT2 model for understanding the use of technology by older adults is largely unknown in existing technology adoption literature, specifically in the healthcare technology adoption domain. This study provides important stride to fill this void in the existing literature by providing empirical evidence. Our analyses of the three different models provide important direction for future researchers in studying technology use behavior.

#### Practical contribution

The study provides valuable information for government and healthcare organizations to improve the adoption of mHealth by older adults in Hong Kong. The findings suggest that if government policymakers could increase the trust level of older adults in mHealth technology, it would enhance the behavior intention for mHealth services usage. Practical implications of the findings are further elaborated below:

- First, mHealth services providers need to increase the trust level of older adults by showing their achievement, skills and reliability as well as improving app design and presenting the advantages of their services better.
- Second, mHealth service providers are urged to employ age-friendly strategies to design mHealth services that can fit older adults with different levels of digital health literacy. It can promote their service quality to establish a trusting relationship between service providers and older users.
- Third, the government should undertake the responsibility to reinforce the safety and effectiveness of mHealth services. The government should formulate regulations to govern the mHealth services providers. This policy indicates that the government supports and monitors the development of mHealth services. It can also enhance older adults’ confidence in adopting mHealth services in their daily life.

#### Limitations of the study

This study has several limitations. First, the findings of this study may not be generalizable to a broader geographical or age population as the source of data was only from older adults (65 years or older) in Hong Kong. Future studies may include a more heterogeneous group of samples from a broader range of ages and geographical regions. The second limitation is that we used self-reported surveys for this study. Although self-reported surveys are popular and widely used by the research community for studying technology adoption research, this method of collecting data is known to contribute to many different biases in the study results [53]. Future studies can be designed to avoid these biases as well as appropriate steps can be taken to mitigate some of these biases. The third limitation of this study comes from the contextualization of a specific technology. This study was conducted in the context of mHealth services, and the results may not be applicable to understanding behavioral intention for using other healthcare technologies and their associated services (e.g., EHR, PHR, etc.). An additional study must be conducted to test the validity of our proposed simplified HTSA model in broader technological, geographical, and population settings. The fourth and final limitation is that this study did not consider health-related variables, such as the health condition or health status of the participants. We acknowledge that different health conditions of older adults could influence their decision on mHealth technology use intention differently; therefore, this should be considered in a future study.

## Conclusion

This study investigated factors to understand behavioral mechanisms for forming the intention to continue to use mHealth services by older adults in Hong Kong. We first analyzed the responses using the original UTAUT2 and our proposed extended UTAUT2 models. The analysis of these two models revealed interesting results. Though our proposed extended model showed better performance than the original UTAUT2 model in explaining behavioral intention to continue to use mHealth services by older adults in Hong Kong, six of the seven factors in the original UTAUT2 were found insignificant in explaining the use behavior of healthcare technology. Only trust and habit showed a positive association with the behavioral intention for the use of mHealth services by older adults.

Based on our findings of the first two models, we further proposed and validated a third model (HTSA model) that is a more simplified version of the extended UTAUT model. The results showed that the HTSA model performed better than the original UTAUT2 and our previously proposed extended models in explaining behavioral intention to continue to use mHealth services by older adults in Hong Kong.

During the test of the HTSA model, it was found that service quality, social influence, and government policy were the strong determinants of trust and insignificant determinants of behavioral intention. Hence, the authors conclude that trust should be used as a mediating factor for the relationships between service quality, social influence, and government policy and behavioral intention. We believe these findings are interesting and novel and provide an in-depth understanding of the formation of healthcare technologies use behavior of older adults, especially in the Hong Kong context.

## Supplementary Information

**Additional file 1.** Survey tools

## Data Availability

The datasets generated and analysed during the current study are not publicly available due to the restrictions involved when obtaining ethical approval for our study, which commit us to sharing the data only with members of the research team but allow data to be made available from the corresponding author upon reasonable request.

## Abbreviations

AVE: Average variance extracted
BI: Behavioral intention
CVI: Content validity index
EE: Effort expectancy
FC: Facilitating conditions
GP: Government policy
HA: Habit
HM: Hedonic motivation
HTMT: Heterotrait-monotrait
HTSA: Healthcare Technology Service Acceptance
PDAs: Personal digital assistants
PE: Performance Expectancy
PLS: Partial least squares
PV: Price value
RA: Research assistant
SEM: Structural Equation Model
SI: Social influence
SMS: Short messaging service
SQ: Service quality
SRMR: Standardized root mean square residual
TAM: Technology acceptance model
TR: Trust
TRA: Theory of reasoned action
UTAUT & UTAUT2: Unified Theory of Acceptance and Use of Technology
VIF: Variance inflation factor
WHO: World Health Organization
