# Supplementary material for: The role of trust and habit in the adoption of mHealth by older adults in Hong Kong: a healthcare technology service acceptance (HTSA) model
Source: BMC Geriatr. 2023 Feb 4;23:73. doi: 10.1186/s12877-023-03779-4 (PMC9898708; doi:10.1186/s12877-023-03779-4)
Supplement: Supplementary file 1 — Additional file 1. Survey tools [file 12877_2023_3779_MOESM1_ESM.docx]

Additional file 1 Survey Tools

Summary of construct with measurement items.

| Constructs | Items | References |
| --- | --- | --- |
| Performance Expectancy (PE) | PE1. I find mHealth services useful in my daily life. |  |
|  | PE2. Using mHealth services helps me accomplish my healthcare activities more quickly. |  |
|  | PE3. Using mHealth services increases my productivity. |  |
|  | PE4. mHealth services help improve the effectiveness of health services. |  |
|  | PE5. mHealth services help save lives. |  |
|  | PE6. mHealth services help provide equitable access to health services. |  |
| Effort Expectancy (EE) | EE1. Learning how to use the mHealth service is easy for me. |  |
|  | EE2. My interaction with the mHealth service is clear and understandable. |  |
|  | EE3. I find the mHealth service easy to use. |  |
|  | EE4. It is easy for me to become skillful at using the mHealth service. |  |
| Facilitating Conditions (FC) | FC1. I have the resources necessary to use an mHealth service. |  |
|  | FC2. I have the knowledge necessary to use an mHealth service. |  |
|  | FC3. mHealth is compatible with other technologies I use. |  |
|  | FC4. I can get help from others when I have difficulties using mHealth. |  |
| Hedonic Motivation (HM) | HM1. Using an mHealth service is fun. |  |
|  | HM 2. Using an mHealth service is enjoyable. |  |
|  | HM 3. Using an mHealth service is very entertaining. |  |
| Social Influence (SI) | SI1. People who are important to me think that I should use an mHealth service. |  |
|  | SI2. People who influence my behavior think that I should use an mHealth service. |  |
|  | SI3. People whose opinions I value prefer that I use an mHealth service. |  |
|  | SI4: If I see people I know are using an mHealth service, it would motivate me to use an mHealth service. |  |
| Price Value (PV | PV1. The mHealth service is reasonably priced. |  |
|  | PV2. The mHealth service offers greater benefit than its cost. |  |
|  | PV3. At the current price, the mHealth service provides good value |  |
| Habit (HA) | HA1. The use of the mHealth service has become a habit for me. |  |
|  | HA2. I must use the mHealth service. |  |
|  | HA3. Using the mHealth service has become natural to me. |  |
| Behavioral Intention (BI) | BI1. I intend to continue using an mHealth service in the future. |  |
|  | BI2. I will always try to use an mHealth service in my daily life. |  |
|  | BI3. I plan to continue to use an mHealth service frequently. |  |
| Service Quality (SQ) | I can access the mHealth service whenever I need to. |  |
|  | SQ2. Physicians at the mHealth platform are competent in providing services. |  |
|  | SQ3. Physicians at the mHealth platform provide me prompt service. |  |
|  | SQ4. Physicians at the mHealth platform gives me personal attention. |  |
|  | SQ5. Physicians at the mHealth platform are willing to help me. |  |
|  | SQ6. I feel safe while consulting with physicians at the mHealth platform. |  |
| Trust (TR) | TR1: Using mHealth services depends on Trust. |  |
|  | TR2: Lack of trust is a barrier to using mHealth technology. |  |
|  | TR3: Trust in the authenticity of information is important in mHealth technology adoption. |  |
|  | TR4: Trust in the reliability of service is important in mHealth technology adoption. |  |
